# Investigation of CNS depressant and muscle relaxant effects of the ethnomedicinal plant *Macropanax dispermus* on Swiss Albino mice and its effect against oxidative stress

**DOI:** 10.5114/bta.2024.141804

**Published:** 2024-09-30

**Authors:** Syeda Rubaiya Afrin, Mohammad Rashedul Islam, Md. Ashraful Alam, Ummah Tasnim Nisat, Bakul Akter, Mohammed Kamrul Hossain

**Affiliations:** 1 Department of Pharmacy, Faculty of Biological Sciences, University of Chittagong, Chittagong, Bangladesh; 2 Department of Pharmacy, Jashore University of Science and Technology, Bangladesh; 3 Department of Pharmacy, University of Science and Technology, Chittagong, Bangladesh

**Keywords:** *Macropanax dispermus*, CNS depressant, muscle relaxant, anxiolytic, antioxidant

## Abstract

**Background and objective:**

Since plant-based natural drugs are widely accepted in modern times and possess numerous pharmacological effects with an extensive therapeutic range, an ethnomedicinal plant native to Bangladesh was selected to investigate for investigation of its various pharmacological effects. *Macropanax dispermus* has been traditionally used and has demonstrated numerous pharmacological effects in preclinical investigations. Therefore, this research aimed to assess the central nervous system (CNS) depressant and antioxidant activities of the crude methanol extracts of the stem barks (MDMS), leaves (MDML), and their different fractions.

**Methods:**

The CNS depressant activity was assessed using the hole cross, rota-rod, and elevated plus maze tests on Swiss Albino mice, while the antioxidant activity was evaluated using the 2,2-diphenyl-1-picrylhydrazyl free radical, hydrogen peroxide (H_2_O_2_) nonradical scavenging, and ferric reducing power assays.

**Results:**

The conducted assays successfully demonstrated that the chloroform fraction of MDML is a significantly (*P* < 0.001) effective CNS depressant, muscle relaxant, and anxiolytic agent with excellent antioxidative effects compared to standard and control. The aqueous fraction of MDML also acted as a significantly (*P* < 0.001) active CNS depressant and muscle relaxant, and it was a highly active ferric-reducing agent. All effects were dose and concentration-dependent.

**Conclusion:**

The presence of various phytochemicals might contribute to these activities. However, further research is suggested to isolate their active compounds and evaluate their mechanisms of action.

## Introduction

Stress is one of the most common problems in the modern world, leading to a variety of psychiatric disorders such as depression, anxiety, epilepsy, schizophrenia, parkinsonism, and restlessness (Sheik et al., 2015). Most prescribed drugs for these disorders, including benzodiazepines, diazepam, zolpidem, zopiclone, and zaleplon, cause serious adverse effects. These can include tiredness, weight gain, nausea, dry mouth, sexual dysfunction, amnesia, sedation, headache, and wooziness (Sheik et al., 2014). Many anxiolytics, muscle relaxants, and sedatives work by depressing the central nervous system (CNS), with benzodiazepines being the most popular. Continuous use of these drugs can lead to addiction, confusion, aggression, excitement, anterograde amnesia, physical dependence, tolerance, and impaired cognitive functioning and psychomotor activity (Kumar and Sharma, 2006). Given these severe adverse effects, significant research has been conducted on various medicinal plants to find safe, effective, and natural treatments for CNS-related disorders.

Free radicals, produced in biological systems and introduced exogenously, can cause various degenerative diseases (Bhattacharya, 2015). Antioxidants combat these free radicals by impeding any of the three major steps of free radical-mediated oxidative processes: initiation, propagation, and termination (Polumbryk et al., 2013). If the balance between reactive oxygen species (ROS) or free radical production during metabolism and antioxidant defenses is lost, “oxidative stress” occurs (Chiurchiù et al., 2016). This stress deregulates cellular functions, leading to diseases such as diabetes mellitus, ischemia/reperfusion injury, inflammatory diseases, cancer, cardiovascular and neurodegenerative ailments, and aging (Valko et al., 2007). Treatment for these diseases is highly expensive and often comes with serious adverse effects. Since natural antioxidants have diverse biochemical activities – including inhibition of ROS, direct or indirect scavenging of free radicals, and alteration of intracellular redox potential – without side effects, they present a promising treatment option for oxidative stress-mediated chronic disorders (Garcia et al., 2012).

Herbal medicine is one of the most popular forms of nature-derived, plant-based traditional medicine, considered an inexpensive remedy for a variety of complicated diseases. Approximately 500 medicinal plants are described in ancient literature, and around 800 plants have been used in traditional medicine (Verma and Singh, 2008). Despite being an age-old system of medicine, its popularity continues to grow due to reduced toxicity and fewer side effects compared to allopathic or synthetic drugs. According to the World Health Organization (WHO), about 70–95% of the world’s population still relies on herbal medicine, especially in developing countries, for primary healthcare (Rahman et al., 2022). This trend is believed to be due to better cultural acceptability, compatibility with the human body, and fewer side effects. Therefore, the present research focuses on developing a medicinal plant-based remedy for anxiety, muscle spasms, sleep disorders, and oxidative stress-mediated diseases.

*Macropanax dispermus*, a tree from the *Araliaceae* family, is an ethnomedicinal plant commonly grown in evergreen forested areas. It is traditionally used by ethnic people of Thailand and Myanmar for treating digestion issues, postpartum bathing, eliminating waste matter, improving blood flow, cough, menopausal fever, and malarial fever (Ong et al., 2018; Panyaphu et al., 2011). Previous studies reported that its crude methanol extracts contain significant amounts of vitamin E, carotene, xanthophylls, tannins, phenolics, and the highest amount of vitamin C, determined using the β-carotene bleaching method (Chanwitheesuk et al., 2005). Additionally, previous investigations have found several phytochemicals, as well as thrombolytic, cytotoxic, analgesic, antipyretic, anti-inflammatory, and antiarthritic activities in MDML, its different fractions, and MDMS (Afrin et al., 2020; Afrin et al., 2021; Afrin et al., 2024). Considering its significant ethnomedicinal uses by tribal people and previously reported research, this study investigates its crude methanolic extracts and their fractions, such as *n* -hexane (MDHL), carbon tetrachloride (MDTL), chloroform (MDCL), ethyl acetate (MDEL), and aqueous fraction (MDAL), to explore its potential in treating anxiety, muscle spasms, and oxidative stress.

## Materials and methods

### Chemicals

Methanol, *n* -hexane, carbon tetrachloride, chloroform, ethyl acetate, and other chemicals used for extraction, solvent–solvent partitioning of plant materials, and *in vitro* and *in vivo* pharmacological tests were of laboratory grade (Merck, Germany).

### Collection and identification of plants

The full-grown plant leaves and stem barks were collected in August 2018 from the Rangamati district (Chattogram Hill Tracts), Chattogram division of Bangladesh, with the assistance of a well-known local traditional healer. The plant was identified as *M. dispermus* by Dr. Shaikh Bokhtear Uddin, a renowned taxonomist and Professor in the Department of Botany at the University of Chittagong, Bangladesh. A specimen was preserved under herbarium no. sr20385.

### Preparation of crude extracts

The plant materials (leaves and stem barks) were washed, cut into small pieces, and dried under semi-shaded sunlight for seven days. After drying, the leaves and stem barks were ground into powder (leaves: 1.36 kg; stem barks: 493 g) and soaked in 7.29 l and 2.60 l of methanol, respectively. After filtration, the filtrate was concentrated using a rotary evaporator (Stuart, UK) under reduced pressure at a temperature below 50°C. The final weights of the obtained crude methanol extracts of *M. dispermus* leaves and stem barks were 28.50 and 7.66 g, respectively. The percentage yield of the extract was calculated using the following equation (Mate et al., 2008):


$$\text{Yield}\;\text{of}\;\text{extracts}\;\left[\%\right]=\frac{\text{Weight}\;\text{of}\;\text{extract}}{\text{Weight}\;\text{of}\;\text{powder}}\times 100$$

The percentage yield of crude methanol extracts of *M. dispermus* leaves and stem barks were 2.09% and 1.55%, respectively.

### Solvent-solvent partitioning

Crude methanol extracts of *M. dispermus* leaves underwent solvent – solvent partitioning according to the protocol designed by Kupchan and Tsou and a modified version by Wagenen et al., using the solvents *n* -hexane, carbon tetrachloride, chloroform, and ethyl acetate consecutively (Kupchan et al., 1973; VanWagenen et al., 1993).

### Experimental animals

Male Swiss Albino mice weighing approximately 20–30 g were used for experimental purposes. They were housed in standard propylene cages and acclimatized under controlled conditions (room temperature of 25 ± 2°C, relative humidity of 60–70%) for 14 days and operated with a 12 h light/dark cycle. The mice were provided with a nutritionally adequate diet and drinking water ad libitum throughout the study. All segments of this report adhere to the ARRIVE Guidelines, version 1 (Kilkenny et al., 2010) for reporting animal research. All experiments were examined and approved by the ethical committee of the Department of Pharmacy, Faculty of Biological Sciences, University of Chittagong, Bangladesh under approval no. cc98056; 10/05/2018.

### Acute toxicity study

An acute toxicity study was conducted following the previously described method (Al-Araby et al., 2020). Each group comprised five Swiss albino mice, which were fasted overnight before extract administration. Each group of animals was administered oral doses of 1000, 2000, 3000, and 4000 mg/kg of body weight of each extract. After the administration of plant extracts, they were restrained from food for a further 3–4 h. Each animal was observed for the first 30 min, then for the first 24 h, and thereafter for 3 days. The mice were monitored for any changes in the skin, fur, eyes, mucous membranes, respiration rate, circulatory rate, and central and autonomic nervous systems at least once a day. The effective dose was determined to be one-tenth of the median lethal dose (LD_50_).

### Study design

During the evaluation of antioxidant activity using the DPPH scavenging assay, 5 mg of each extract was dissolved in 10 ml of methanol to prepare a stock solution of 500 μg/ml. These stock solutions were serially diluted to 250, 125, 62.5, 31.25, and 15.63 μg/ml with methanol. In the hydrogen peroxide scavenging assay and reducing power assay, distilled water was used for serial dilution instead of methanol, following the same procedure. For CNS depressant activity, 16 groups of mice were used for each investigation, with five mice in each group. Group I served as the control (1% Tween-80, 10 ml/kg), Group II received the standard treatment (Diazepam 1 mg/kg), and the remaining groups were administered MDMS, MDML, and their different extracted fractions at doses of 200 and 400 mg/kg. After each experimental period, all treated mice were sacrificed using diethyl ether anesthesia.

### CNS depressant activity

#### Hole cross test

The experiment was conducted using a previously reported method (Hussain et al., 2012). After administering the test samples to each group of mice, they were placed in the hole cross apparatus (dimensions: 30 × 20 × 14 cm, hole size: 7.5 × 3 cm) and allowed to move through the hole from one chamber to another spontaneously. The number of passages was recorded before the treatment and at 30, 60, 90, and 120 min after the treatment for 3 min. The percentage inhibition of movements was calculated using the following formula:


$$\text{Movements}\;\text{inhibition}\;\text{[\%]}=\frac{\text{No.}\;\text{of}\;\text{movement}\;\text{(contr.) }-\text{ No.}\;\text{of}\;\text{movement}\;\text{(test)}}{\text{No.}\;\text{of}\;\text{movement}\;\text{(contr.)}}\times 100$$

#### Rota-rod test

The rota-rod test was performed using a rota-rod apparatus consisting of a circular, horizontal nonslippery rotating rod turning at a constant or increasing speed (20 RPM), according to the procedure (Bohlen et al., 2009). Each group of mice was trained one day before the experiment. Mice that could remain on the rotating rod for 180 s or longer were selected. After 30 min of the administration of test samples, each group of mice was placed on the rod for 180 s, and the falling time for each mouse from the rotating rod was recorded. The percentage reduction in falling time was calculated as follows:


$$\text{Reduction}\;\text{in}\;\text{fall}\;\text{off}\;\text{time}\;\text{[\%]}=\frac{A-B}{\text{A}}\times 100$$

Here, *A* – fall off time pretreatment, *B* – fall off time posttreatment.

#### Elevated plus maze test

The study was conducted according to a previously reported method (Emon et al., 2020). After 30 min of administration of test samples, each animal was placed in the center of the maze, with its head facing towards the open arm, and the following parameters were recorded for 3 min: 1) the first preference of the mouse to the open or closed arm, 2) the number of entries into the open and closed arms, and 3) the time each animal spent in the open and closed arms. These observations were recorded for each animal in each group and compared (SK 2009).

### Antioxidant activity

#### DPPH scavenging assay

The 2,2-diphenyl-1-picrylhydrazyl (DPPH) free radical scavenging activity of the prepared test samples was measured according to a previously described method with slight modifications (Bitalebi et al., 2019). The working solution was prepared by diluting a 0.004% w/v DPPH solution with methanol to obtain an absorbance of about 0.98 ± 0.02 at 517 nm using a UV-Vis spectrophotometer. A 3 ml portion of this solution was mixed with 2 ml of the prepared DPPH solution as the negative control, ascorbic acid (AA) as the positive control, and the test samples at different concentrations (15.63–500 μg/ml), followed by incubation in the dark for 30 min at room temperature. Then, the absorbance was measured at 517 nm using a UV–Vis spectrophotometer. The percentage of DPPH scavenging activity was calculated using the following equation:


$$\text{Scavenging}\;\text{effect}\;\text{(DPPH)}\;\text{[\%]}=\frac{\text{Absorbance}\;\text{of}\;\text{control}-\text{Absorbance}\;\text{of}\;\text{sample}}{\text{Absorbance}\;\text{of}\;\text{control}}\times 100$$

#### Hydrogen peroxide scavenging assay

The hydrogen peroxide scavenging assay was conducted using a previously described method with slight modifications (Al-Amiery et al., 2015). A 2 mM hydrogen peroxide (H_2_O_2_) solution was prepared in 50 mM phosphate buffer (pH 7.4), and its concentration was determined at 230 nm using a UV–Vis spectrophotometer. Aliquots of 0.1 ml of 50 mM phosphate buffer without H_2_O_2_ served as the negative control, AA as the positive control, and test samples at different concentrations (15.63–500 μg/ml) were mixed with 50 mM phosphate buffer (pH 7.4) up to 0.4 ml. After adding 0.6 ml of the hydrogen peroxide solution, the mixtures were vortexed, and the absorbance of hydrogen peroxide at 230 nm was determined after 10 min, against a blank solution. The ability to scavenge hydrogen peroxide was calculated using the following equation (Gülçin et al., 2004):


$$\text{Scavenging}\;\text{effect}\;{\text{(H}}_{2}{\text{O}}_{2}\text{)}\;\text{[\%]}=\frac{\text{Absorbance}\;\text{of}\;\text{control}-\text{Absorbance}\;\text{of}\;\text{sample}}{\text{Absorbance}\;\text{of}\;\text{control}}\times 100$$

#### Reducing power assay

The reducing power assay was conducted based on the principle of transforming Fe (III) to Fe (II) in the presence of test samples (Li et al., 2020). Two milliliters of test samples at different concentrations (15.63–500 μg/ml) were mixed with 2 ml of phosphate buffer (0.2 M, pH 6.6) and 2 ml of 1% potassium ferricyanide. The mixture was incubated at 50°C for 20 min, followed by the addition of 2 ml of 100 mg/l trichloroacetic acid. The mixture was then centrifuged at 3000 rpm for 10 min. The collected upper layer was mixed with 2 ml of distilled water and 0.4 ml of 0.1% w/v freshly prepared ferric chloride solution. After 10 min of reaction, the absorbance was measured at 700 nm. The percentage of reducing power was calculated using the following formula:


$$\text{Reducing}\;\text{power}\;\text{[\%]}=\frac{\text{Absorbance}\;\text{of}\;\text{sample}-\text{Absorbance}\;\text{of}\;\text{control}}{\text{Absorbance}\;\text{of}\;\text{sample}}\times 100$$

### Determination of median inhibitory concentration (IC_50_)

The median inhibitory concentration (IC_50_) represents the concentration of the sample required to inhibit 50% of the DPPH and hydrogen peroxide after a certain exposure time. It was determined by the linear regression method by plotting the percentage of the scavenging effect against the corresponding logarithm of concentration. An approximate linear correlation was observed on the graph paper, and the concentration-scavenging data were transformed into a straight line using trend line fit linear regression analysis (Microsoft Excel, 2007); the IC_50_ was derived from the best-fit line obtained.

### Statistical analysis

All the data were expressed as mean ± SEM (standard error of the mean). The results were analyzed statistically by one-way ANOVA followed by posthoc Dunnett’s test using “Statistical Package for Social Science” (SPSS, Version 16.0, IBM Corporation, NY). Results with *P < 0.05, **P < 0.01, and ***P < 0.001 were considered statistically significant compared to the control.

## Results

### CNS depressant activity

#### Hole cross test

In this test, a decrease in the movement of the mice through the hole indicates the gradual depression of the CNS. The present research demonstrated that the control did not significantly inhibit the movement of mice, whereas the Diazepam standard significantly inhibited movement from 46.67 to 94.74% (*P* < 0.001). MDAL exhibited a higher percentage of movement inhibition at both doses, with 28.33 to 91.23% (200 mg/kg) and 38.33 to 92.98% (400 mg/kg) at different time intervals. MDHL, MDTL, MDCL, and MDEL also significantly decreased the number of movements in a dose-dependent manner, demonstrating their potential as strong CNS depressant agents. MDMS was observed to show a lower percentage of movement inhibition at both doses, with 18.33 to 82.46% (200 mg/kg) and 45 to 84.21% (400 mg/kg) at different time intervals. All these results are shown in Table 1.

**Table 1:** Screening of CNS depressant activity of MDML, its solvent fraction, and MDMS stem barks by calculating the mean number of movements of mice in hole cross test

Group	Dose [mg/kg]	Number of movements of mice (% inhibition of movements)				
		pretreatment	30 min	60 min	90 min	120 min
Control	–	13.4 ± 1.5	12 ± 1.41	11.4 ± 1.36	11.2 ± 1.16	11.4 ± 1.03
Diazepam	1	14.2 ± 1.77	6.4 ± 1.25* (46.67%)	3.6 ± 0.81*** (68.42%)	1 ± 0.32*** (91.07%)	0.6 ± 0.24*** (94.74%)
MDML	200	15 ± 1.22	7 ± 1.3* (41.67%)	5.4 ± 0.68*** (52.63%)	2.2 ± 0.37*** (80.36%)	1.4 ± 0.51*** (87.72%)
	400	11.2 ± 1.2	5.4 ± 0.6** (55%)	4.2 ± 0.49*** (63.15%)	2.2 ± 0.2*** (80.36%)	1.2 ± 0.37*** (89.47%)
MDHL	200	12.6 ± 1.5	10.2 ± 1.43 (15%)	6.2 ± 0.97** (45.61%)	3.4 ± 0.87*** (69.64%)	1.6 ± 0.51*** (85.96%)
	400	12 ± 0.95	7.6 ± 0.75 (36.67%)	5.4 ± 0.81*** (52.63%)	1.4 ± 0.6*** (87.5%)	1 ± 0.45*** (91.23%)
MDTL	200	14.8 ± 0.66	9 ± 0.89 (25%)	6.6 ± 0.24** (42.11%)	3.8 ± 0.49*** (66.07%)	3 ± 0.55*** (73.68%)
	400	15.2 ± 1.16	7.2 ± 1.07 (40%)	4.8 ± 0.8*** (57.89%)	1.8 ± 0.58*** (83.93%)	1 ± 0.45*** (91.23%)
MDCL	200	13.6 ± 1.21	9.6 ± 1.36 (20%)	4.8 ± 0.97*** (57.89%)	3 ± 0.71*** (73.21%)	1.6 ± 0.51*** (85.96%)
	400	15.6 ± 1.08	8 ± 1.41 (33.33%)	4.2 ± 0.97*** (63.16%)	1.2 ± 0.58*** (89.29%)	0.6 ± 0.24*** (94.74%)
MDEL	200	13.4 ± 1.21	9 ± 0.95 (25%)	6 ± 0.95*** (47.37%)	2.6 ± 0.51*** (76.79%)	1.8 ± 0.37*** (84.21%)
	400	16.2 ± 1.53	8.6 ± 1.81 (28.33%)	3.6 ± 0.68*** (68.42%)	1 ± 0.55*** (91.07%)	0.6 ± 0.4*** (94.74%)
MDAL	200	16 ± 1.22	8.6 ± 1.03 (28.33%)	4.6 ± 0.98*** (59.65%)	2.2 ± 0.37*** (80.36%)	1 ± 0.45*** (91.23%)
	400	16.2 ± 1.24	7.4 ± 1.12 (38.33%)	3.2 ± 0.87*** (71.93%)	1.8 ± 0.58*** (83.93%)	0.8 ± 0.37*** (92.98%)
MDMS	200	14.6 ± 1.82	9.8 ± 0.66 (18.33%)	6.6 ± 0.75** (42.11%)	3.6 ± 0.51*** (67.86%)	2 ± 0.45*** (82.46%)
	400	13.6 ± 1.57	6.66 ± 0.93* (45%)	4.2 ± 0.73*** (63.16%)	2.4 ± 0.4*** (78.57%)	1.8 ± 0.58*** (84.21%)

MDML – crude methanol extract of *M. dispermus* leaves, MDHL – *n* -hexane fraction of crude methanol extract of *M. dispermus* leaves, MDTL – carbon tetrachloride fraction of crude methanol extract of *M. dispermus* leaves, MDCL – chloroform fraction of crude methanol extract of *M. dispermus* leaves, MDEL – ethyl acetate fraction of crude methanol extract of *M. dispermus* leaves, MDAL – aqueous fraction of crude methanol extract of *M. dispermus* leaves, MDMS – crude methanol extract of *M. dispermus* stem barks; results were expressed as mean ± SEM;

* *P* < 0.05,

** *P* < 0.01,

*** *P* < 0.001 were considered statistically significant compared to control

#### Rota-rod test

**Fig. 1:**
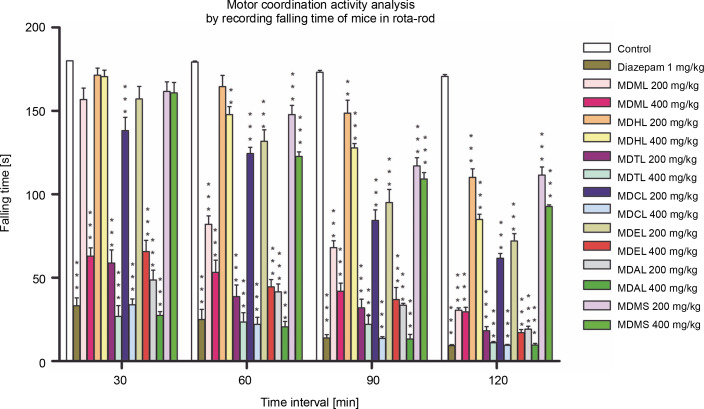
Screening of CNS depressant activity of MDML, its solvent fraction and MDMS by calculating mean falling time of mice in rotarod; MDML – crude methanol extract of *M. dispermus* leaves, MDHL – *n* -hexane fraction of crude methanol extract of *M. dispermus* leaves, MDTL – carbon tetrachloride fraction of crude methanol extract of *M. dispermus* leaves, MDCL – chloroform fraction of crude methanol extract of *M. dispermus* leaves, MDEL – ethyl acetate fraction of crude methanol extract of *M. dispermus* leaves, MDAL – aqueous fraction of crude methanol extract of *M. dispermus* leaves, MDMS – crude methanol extract of *M. dispermus* stem barks; results were expressed as mean ± SEM; *P < 0.05, **P < 0.01, ***P < 0.001 were considered statistically significant compared to control

In this procedure, a muscle-relaxing agent will cause a decrease in the falling time of mice from the rota-rod as time passes. The current study showed that the control group exhibited a very low and gradual increase in the percentage reduction of falling time from the rotarod (0 to 5.16%), whereas the reference standard showed the highest and most gradual increase in the percentage reduction of falling time (81.56 to 94.82%) significantly (*P* < 0.001). MDAL significantly reduced the fall-off time from the rotarod (*P* < 0.001) at different time intervals, ranging from 72.96 to 83.26% at 200 mg/kg and from 84.75 to 95.61% at 400 mg/kg. These data are presented in Table 2. MDTL, MDCL, and MDEL were also found to significantly decrease the falling time from the rotarod, indicating a higher muscle relaxant effect (Fig. 1). MDMS exhibited the least muscle relaxant effect, with a reduction in falling time ranging from 10.18 to 37.99% (200 mg/kg) and from 10.67 to 48.47% (400 mg/kg).

**Table 2:** CNS depressant activity of the investigated extracts of *M. dispermus* leaves and stem barks by using the rotarod test

Group	Dose [mg/kg]	Falling time of mice in seconds (% of reduction in falling time)				
		pretreatment	30 min	60 min	90 min	120 min
Control	–	180 ± 0	180 ± 0 (0%)	179.316 ± 1.42 (0.38%)	173.168 ± 1.14 (3.8%)	170.72 ± 1.11 (5.16%)
Diazepam	1	180 ± 0	33.188 ± 4.75*** (81.56%)	24.938 ± 6.17*** (86.15%)	13.928 ± 2.06*** (92.26%)	9.322 ± 0.65*** (94.82%)
MDML	200	180 ± 0	156.83 ± 6.84 (12.87%)	82.052 ± 4.95*** (54.42%)	68.08 ± 4.16*** (62.18%)	30.538 ± 1.4*** (83.03%)
	400	180 ± 0	62.896 ± 5.08*** (65.06%)	53.238 ± 7.37*** (70.42%)	41.904 ± 4.92*** (76.72%)	29.602 ± 2.66*** (83.55%)
MDHL	200	180 ± 0	171.4 ± 4.25 (4.78%)	164.524 ± 6.73 (8.6%)	148.726 ± 7.67** (17.37%)	110. 226 ± 5.14*** (38.76%)
	400	180 ± 0	170.542 ± 3.85 (5.25%)	147.83 ± 4.73** (17.87%)	127.822 ± 2.64*** (28.99%)	84.926 ± 3.18*** (52.82%)
MDTL	200	180 ± 0	58.83 ± 7.83*** (67.32%)	38.664 ± 7.01*** (78.52%)	31.92 ± 5.33*** (82.27%)	18.152 ± 2.5*** (89.92%)
	400	180 ± 0	26.81 ± 6.52*** (85.11%)	23.444 ± 5.67*** (86.98%)	22.068 ± 5.34*** (87.74%)	11.02 ± 0.85*** (93.88%)
MDCL	200	180 ± 0	138.234 ± 7.9*** (23.2%)	124.478 ± 3.72*** (30.85%)	84.364 ± 6.35*** (53.13%)	61.662 ± 2.92*** (65.74%)
	400	180 ± 0	33.84 ± 3.49*** (81.2%)	22.09 ± 4.19*** (87.72%)	13.67 ± 0.96*** (92.41%)	9.566 ± 0.45*** (94.69%)
MDEL	200	180 ± 0	157.214 ± 7.45 (12.66%)	131.798 ± 6.9*** (26.78%)	95.114 ± 7.78*** (47.16%)	72.014 ± 4.46*** (59.99%)
	400	180 ± 0	65.706 ± 6.7*** (63.5%)	44.5 ± 4.43*** (75.28%)	36.974 ± 7.26*** (79.46%)	17.12 ± 1.83*** (90.49%)
MDAL	200	180 ± 0	48.67 ± 5.92*** (72.96%)	41.612 ± 4.74*** (76.88%)	33.548 ± 1.17*** (81.36%)	19.148 ± 1.8*** (89.36%)
	400	180 ± 0	27.442 ± 2.25*** (84.75%)	20.558 ± 3.25*** (88.58%)	13.344 ± 2.7*** (92.59%)	9.698 ± 0.98*** (94.61%)
MDMS	200	180 ± 0	161.672 ± 5.72 (10.18%)	147.752 ± 5.61*** (17.92%)	117.032 ± 4.85*** (34.98%)	111.606 ± 4.87*** (37.99%)
	400	180 ± 0	161.802 ± 6.24 (10.67%)	122.662 ± 2.76*** (31.85%)	109.154 ± 3.88*** (39.36%)	92.748 ± 0.9*** (48.47%)

MDML – crude methanol extract of *M. dispermus* leaves, MDHL – *n* -hexane fraction of crude methanol extract of *M. dispermus* leaves, MDTL – carbon tetrachloride fraction of crude methanol extract of *M. dispermus* leaves, MDCL – chloroform fraction of crude methanol extract of *M. dispermus* leaves, MDEL – ethyl acetate fraction of crude methanol extract of *M. dispermus* leaves, MDAL – aqueous fraction of crude methanol extract of *M. dispermus* leaves, MDMS – crude methanol extract of *M. dispermus* stem barks; results were expressed as mean ± SEM; *P < 0.05,

** *P* < 0.01,

*** *P* < 0.001 were considered statistically significant compared to control

#### Elevated plus maze test

**Fig. 2:**
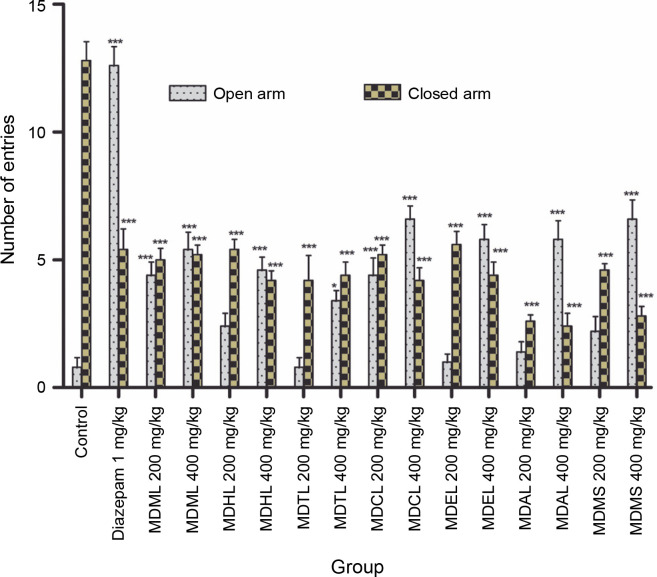
Screening of anxiolytic activity of MDML, its solvent fraction, and MDMS by counting the mean number of entries of mice into the open and closed arms on the elevated plus maze

In this research, control mice entered the closed arm 12.8 ± 0.73 times, spent most of the experimental time in it (177.466 ± 0.69 s), and entered the open arm 0.8 ± ± 0.36 times, spending very little time there (0.406 ± ± 0.25 seconds). In contrast, the reference standard Diazepam, a highly active anxiolytic, showed the reverse results, entering the open arm 12.6 ± 0.75 times (61.896 ± ± 1.43 s) and the closed arm 5.4 ± 0.81 times (115.972 ± ± 1.35 s) significantly (Table 3). When compared to the control, MDCL demonstrated a higher number of entries and more time spent in the open arms but still spent a significant amount of time in the closed arms at both doses. MDTL and MDAL spent much less time in the open arm compared to other extracts and more time in the closed arm (Fig. 2).

**Table 3:** CNS depressant activity of the investigated extracts of *M. dispermus* leaves and stem barks by using the elevated plus maze test

Group	Dose [mg/kg]	Open arm (OA)		Closed arm (CA)	
		time spent	no. of entries	time spent	no. of entries
Control	–	0.406 ± 0.25	0.8 ± 0.37	177.466 ± 0.69	12.8 ± 0.73
Diazepam	1	61.896 ± 1.43***	12.6 ± 0.75***	115.972 ± 1.35***	5.4 ± 0.81***
MDML	200	19.246 ± 1.44***	4.4 ± 0.51***	151.718 ± 1.51***	5 ± 0.45***
	400	23.156 ± 2.63***	5.4 ± 0.68***	148.574 ± 1.46***	5.2 ± 0.37***
MDHL	200	9.46 ± 3.27**	2.4 ± 0.51	166.624 ± 2.81***	5.4 ± 0.4***
	400	26.608 ± 0.98***	4.6 ± 0.51***	147.636 ± 1.45***	4.2 ± 0.37***
MDTL	200	0.95 ± 0.9	0.8 ± 0.37	175.942 ± 1.54	4.2 ± 0.97***
	400	8.328 ± 0.8**	3.4 ± 0.4*	165.75 ± 1.39***	4.4 ± 0.51***
MDCL	200	21.846 ± 1.56***	4.4 ± 0.68***	155.478 ± 1.51***	5.2 ± 0.37***
	400	36.87 ± 0.97***	6.6 ± 0.51***	140.048 ± 0.65***	4.2 ± 0.49***
MDEL	200	2.098 ± 0.57	1 ± 0.32	177.11 ± 0.88	5.6 ± 0.51***
	400	54.62 ± 1.69***	5.8 ± 0.58***	120.546 ± 2.04***	4.4 ± 0.51***
MDAL	200	2.396 ± 0.68	1.4 ± 0.4	174.818 ± 1.21	2.6 ± 0.24***
	400	10.262 ± 1.09***	5.8 ± 0.73***	166.748 ± 1.58***	2.4 ± 0.51***
MDMS	200	5.882 ± 1.46	2.2 ± 0.58	170.11 ± 1.4	4.6 ± 0.24***
	400	33.722 ± 2.27***	6.6 ± 0.75***	143.254 ± 1.71***	2.8 ± 0.37***

MDML – crude methanol extract of *M. dispermus* leaves, MDHL – *n* -hexane fraction of crude methanol extract of *M. dispermus* leaves, MDTL – carbon tetrachloride fraction of crude methanol extract of *M. dispermus* leaves, MDCL – chloroform fraction of crude methanol extract of *M. dispermus* leaves, MDEL – ethyl acetate fraction of crude methanol extract of *M. dispermus* leaves, MDAL – aqueous fraction of crude methanol extract of *M. dispermus* leaves, MDMS – crude methanol extract of *M. dispermus* stem barks; results were expressed as mean ± SEM;

* *P* < 0.05,

** *P* < 0.01,

*** *P* < 0.001 were considered statistically significant compared to control

### Antioxidant activity

#### DPPH scavenging assay

In this assay, MDCL, MDEL, and MDML showed IC_50_ values of 0.35, 8.41, and 67.75 μg/ml, respectively, indicating higher antioxidant activity compared to other extracts and the standard (IC_50_ value of 0.02 μg/ml). MDTL had the highest IC_50_ value of 49082.26 μg/ml, thus possessing the lowest capability to inhibit free radical-mediated oxidation (Table 4).

**Table 4:** Antioxidant activity of the investigated extracts of *M. dispermus* leaves and stem barks by using DPPH scavenging assay

Group	Equation	*_R_*2	IC_50_ [μg/ml]
Control	–	–	**–**
Standard (AA)	*y* = 10.41*x* + 68.39	*R* ^2^ = 0.980	0.02
MDML	*y* = 17.27*x* + 18.38	*R* ^2^ = 0.985	67.75
MDHL	*y* = 29.84*x* − 22.87	*R* ^2^ = 0.916	276.71
MDTL	*y* = 11.79*x* − 5.306	*R* ^2^ = 0.890	49082.26
MDCL	*y* = 14.05*x* + 56.43	*R* ^2^ = 0.944	0.35
MDEL	*y* = 19.07*x* + 32.36	*R* ^2^ = 0.892	8.41
MDAL	*y* = 8.249*x* + 25.96	*R* ^2^ = 0.951	820.90
MDMS	*y* = 24.00*x* − 2.814	*R* ^2^ = 0.857	158.70

AA – ascorbic acid, MDML – crude methanol extract of *M. dispermus* leaves, MDHL – *n* -hexane fraction of crude methanol extract of *M. dispermus* leaves, MDTL – carbon tetrachloride fraction of crude methanol extract of *M. dispermus* leaves, MDCL – chloroform fraction of crude methanol extract of *M. dispermus* leaves, MDEL – ethyl acetate fraction of crude methanol extract of *M. dispermus* leaves, MDAL – aqueous fraction of crude methanol extract of *M. dispermus* leaves, MDMS – crude methanol extract of *M. dispermus* stem bark

#### Hydrogen peroxide scavenging assay

In this method, MDTL, MDCL, and MDEL exhibited higher antioxidant activity compared to other extracts and the standard (IC_50_ value 1.09 μg/ml), with IC_50_ values of 80.17, 112.53, and 160.83 μg/ml, respectively. MDMS had the highest IC_50_ value of 48655.24 μg/ml, indicating the least antioxidant activity (Table 5).

**Table 5:** Antioxidant activity of the investigated extracts of *M. dispermus* leaves and stem barks by using hydrogen peroxide scavenging assay

Group	Equation	*_R_*2	IC_50_ [μg/ml]
Control	–	–	–
Standard (AA)	*y* = 14.89*x* + 49.46	*R* ^2^ = 0.969	1.09
MDML	*y* = 22.25*x* − 21.48	*R* ^2^ = 0.876	1631.49
MDHL	*y* = 29.62*x* − 33.51	*R* ^2^ = 0.885	659.75
MDTL	*y* = 33.54*x* − 13.86	*R* ^2^ = 0.992	80.17
MDCL	*y* = 37.06*x* − 26.02	*R* ^2^ = 0.915	112.53
MDEL	*y* = 37.88*x* − 44.57	*R* ^2^ = 0.9	313.74
MDAL	*y* = 51.46*x* − 63.54	*R* ^2^ = 0.907	160.83
MDMS	*y* = 10.80*x* − 0.621	*R* ^2^ = 0.952	48655.24

AA – ascorbic acid, MDML – crude methanol extract of *M. dispermus* leaves, MDHL – *n* -hexane fraction of crude methanol extract of *M. dispermus* leaves, MDTL – carbon tetrachloride fraction of crude methanol extract of *M. dispermus* leaves, MDCL – chloroform fraction of crude methanol extract of *M. dispermus* leaves, MDEL – ethyl acetate fraction of crude methanol extract of *M. dispermus* leaves, MDAL – aqueous fraction of crude methanol extract of *M. dispermus* leaves, MDMS – crude methanol extract of *M. dispermus* stem bark

#### Reducing power assay

In this method, a higher percentage of reducing capacity indicates higher antioxidant activity of an extract. The standard showed the highest antioxidant activity with a reducing power ranging from 92.48 to 94.44%, in a concentration-dependent manner. MDTL (76.33–90.52%), MDCL (2.44–84.62%), and MDAL (2.44–77.78%) exhibited higher antioxidant activity compared to other extracts and the standard, in a concentration-dependent manner. MDML and MDMS showed the lowest reducing capacity, with the higher concentration of the sample exhibiting 16.67% reducing power (Table 6).

**Table 6:** Antioxidant activity of the investigated extracts of *M. dispermus* leaves and stem barks by using reducing power assay

Group	Percent of reducing power [%]	Group	Percent of reducing power [%]
Control	–	Control	–
AA (500 μg/ml)	94.44	MDCL (500 μg/ml)	84.62
AA (250 μg/ml)	94.04	MDCL (250 μg/ml)	42.03
AA (125 μg/ml)	93.7	MDCL (125 μg/ml)	41.18
AA (62.5 μg/ml)	93.32	MDCL (62.5 μg/ml)	31.03
AA (31.25 μg/ml)	92.88	MDCL (31.25 μg/ml)	21.57
AA (15.63 μg/ml)	92.48	MDCL (15.63 μg/ml)	2.44
MDML (500 μg/ml)	16.67	MDEL (500 μg/ml)	53.49
MDML (250 μg/ml)	14.89	MDEL (250 μg/ml)	42.86
MDML (125 μg/ml)	9.09	MDEL (125 μg/ml)	42.03
MDML (62.5 μg/ml)	4.76	MDEL (62.5 μg/ml)	20
MDML (31.25 μg/ml)	2.44	MDEL (31.25 μg/ml)	13.04
MDML (15.63 μg/ml)	0	MDEL (15.63 μg/ml)	4.76
MDHL (500 μg/ml)	71.22	MDAL (500 μg/ml)	77.78
MDHL (250 μg/ml)	55.06	MDAL (250 μg/ml)	28.57
MDHL (125 μg/ml)	45.95	MDAL (125 μg/ml)	16.67
MDHL (62.5 μg/ml)	45.3	MDAL (62.5 μg/ml)	9.1
MDHL (31.25 μg/ml)	39.4	MDAL (31.25 μg/ml)	6.98
MDHL (15.63 μg/ml)	27.27	MDAL (15.63 μg/ml)	2.44
MDTL (500 μg/ml)	90.52	MDMS (500 μg/ml)	16.67
MDTL (250 μg/ml)	89.9	MDMS (250 μg/ml)	16.67
MDTL (125 μg/ml)	89.25	MDMS (125 μg/ml)	14.89
MDTL (62.5 μg/ml)	88.76	MDMS (62.5 μg/ml)	13.04
MDTL (31.25 μg/ml)	87.84	MDMS (31.25 μg/ml)	9.1
MDTL (15.63 μg/ml)	76.33	MDMS (15.63 μg/ml)	4.76

AA – ascorbic acid, MDML – crude methanol extract of *M. dispermus* leaves, MDHL – *n* -hexane fraction of crude methanol extract of *M. dispermus* leaves, MDTL – carbon tetrachloride fraction of crude methanol extract of *M. dispermus* leaves, MDCL – chloroform fraction of crude methanol extract of *M. dispermus* leaves, MDEL – ethyl acetate fraction of crude methanol extract of *M. dispermus* leaves, MDAL – aqueous fraction of crude methanol extract of *M. dispermus* leaves, MDMS – crude methanol extract of *M. dispermus* stem bark

## Discussion

Herbal medicine has always paved the way for toxicity-free, effective natural remedies for various complicated diseases. The ethnomedicinal plant *M. dispermus* was selected to study its various pharmacological effects. Previous research identified several phytochemicals and various pharmacological effects in MDML, its different fractions, and MDMS, prompting this research to assess its efficacy as a CNS depressant, muscle relaxant, anxiolytic agent, and antioxidant (Afrin et al., 2020; Afrin et al., 2021).

The CNS depressant activity of the plant extracts was explored by estimating locomotion, muscle relaxation or motor coordination activity, and anxiolytic activity using three different methods. In the hole cross test, a mouse was allowed to cross through a hole from one chamber to another on a specially constructed apparatus. The number of movements of mice at different time intervals indicated the locomotor activity. A decrease in locomotion specified the CNS depressant activity of an extract. MDAL, MDHL, MDTL, MDCL, and MDEL significantly decreased the number of movements of mice in a dose-dependent manner compared to the control, suggesting their effectiveness as CNS depressant agents, while MDMS showed the least effectiveness in this method.

In the Rota-rod test, the reduction in falling time of mice from the rotating rod of the designated apparatus due to loss of grip implies skeletal muscle relaxation. This effect also indicates an induced neurological deficit, including a calming effect, which further depicts its CNS depressant effect. In this study, MDAL, MDTL, MDCL, and MDEL were researched to decrease the fall-off time of mice from the apparatus compared to the control. Hence, they could act as potential muscle-relaxing agents. Among all the extracts, MDMS was found to be the least potent muscle-relaxing agent.

In the EPM test, natural stimuli (fear of a novel open space and fear of balancing on a relatively narrow, raised platform) are used to induce anxiety in mice (Dawson and Tricklebank, 1995). The increase in the time spent in the OA and the number of entries into the OA were indices of increased motor activity, hence the anxiolytic effect. In the present research, MDCL demonstrated a higher number of OA entries with significantly increased time spent in the OA compared to the control and standard. Therefore, it could act as a potential anxiolytic agent. On the contrary, MDTL and MDAL illustrated a smaller number of OA entries with less time spent in the OA compared to the control. All of their activities were dose-dependent.

In the current study, the antioxidant activity was assessed using three different methods to justify the antioxidant capacity of different extracts. DPPH is a stable free radical at room temperature and accepts an electron or hydrogen radical to become a stable diamagnetic molecule (Franyoto et al., 2018). This capability was used to evaluate the antioxidant activity of the plant extracts, and the reaction of the sample with DPPH in methanol facilitated the extraction of antioxidant compounds from the sample. In the H_2_O_2_ scavenging assay, the antioxidant compounds in the extracts acted as catalase to donate electrons to a stable, nonradical oxidant H_2_O_2_ and neutralize it into water. Another method used the capability of the antioxidant compounds in extracts to reduce the ferric cyanide complex (Fe^3+^) to the ferrous cyanide form (Fe^2+^). The ROS scavenging method dictated that a lower IC_50_ value is an indicator of higher DPPH and H_2_O_2_ scavenging capacity, hence higher antioxidant activity.

In the current study, MDCL, MDEL, and MDML were observed to be potent free radical scavengers, whereas MDTL had almost no potential to scavenge the free radicals compared to the standard and other extracts. In contrast, MDTL was an effective H_2_O_2_ scavenger with a higher reducing capacity than the standard. MDCL and MDEL showed higher H_2_O_2_ scavenging effects, while MDMS showed almost no H_2_O_2_ scavenging effect or ferric-reducing capacity. MDCL and MDAL were also found to be effective reducing agents. All of these extracts demonstrated their effects in a concentration-dependent manner.

However, in the case of CNS depressant activity, since the standard Diazepam acts by directly activating the gamma-aminobutyric acid (GABA) receptor, it can be predicted that the investigated CNS depressant agents might act by potentiating GABAergic inhibition in the CNS via membrane hyperpolarization or by directly activating the GABA receptors. Several reports have demonstrated that plants and plant extracts rich in alkaloids, glycosides, and flavonoids possess CNS depressant properties, mediated through their affinity (*in vitro* ) with the benzodiazepine site of the GABAergic complex system, or as direct or indirect modulators of this receptor (Awad et al., 2009; Estrada-Reyes et al., 2010; Fernández et al., 2004; Kahnberg et al., 2002; Trofimiuk et al., 2005). Additionally, nonspecific CNS depression can also be attributed to tannins (Takahashi et al., 1986). Therefore, the presence of flavonoids and glycosides might contribute to the CNS depressant activity of MDCL and MDAL.

In addition, the antioxidant activities of assumed antioxidants have been recognized to operate through various mechanisms, including the prevention of chain initiation, binding of transition metal ion catalysts, decomposition of peroxides, prevention of continued hydrogen abstraction, and radical scavenging (Diplock, 1997). Hence, it can be suggested that there is no linear correlation among free radical scavenging, H_2_O_2_ scavenging, and/or reducing power activity. Thus, although MDTL exhibited no free radical scavenging activity, it did show H_2_O_2_ scavenging and reducing power activity.

## Conclusion

In conclusion, MDCL was observed to be a potential CNS depressant, muscle relaxant, and anxiolytic agent. It is also an effective antioxidant, capable of acting through both radical and nonradical scavenging mechanisms or by reducing Fe^3+^ to Fe^2+^. MDEL is an effective DPPH and H_2_O_2_ scavenger, while MDAL is solely an effective reducing agent. Both extracts, MDEL and MDAL, are potential CNS depressants and muscle relaxants. Since MDCL, MDEL, and MDAL exhibit both antioxidant and CNS depressant effects, it is possible that these extracts could be potential drugs for the treatment of oxidative stress-induced neurodegenerative diseases. Further research must be conducted to isolate and identify the active compounds in the investigated extracts and elucidate their possible mechanisms of action using a reasonable kinetic model.
